# Prenatal over‐ and undernutrition differentially program small intestinal growth, angiogenesis, absorptive capacity, and endocrine function in sheep

**DOI:** 10.14814/phy2.14498

**Published:** 2020-06-29

**Authors:** Prabhat Khanal, Anne Marie D. Axel, Sina Safayi, Vibeke S. Elbrønd, Mette O. Nielsen

**Affiliations:** ^1^ Faculty of Biosciences and Aquaculture Animal Science, Production and Welfare Division Nord University Steinkjer Norway; ^2^ Department of Veterinary and Animal Sciences Faculty of Health and Medical Sciences University of Copenhagen Frederiksberg Denmark; ^3^ Graduate College Rush University Chicago IL USA; ^4^ Department of Animal Science Faculty of Technical Sciences Aarhus University Tjele Denmark

**Keywords:** absorption, angiogenesis, intestinal development, postnatal overfeeding, prenatal malnutrition

## Abstract

The aim was to test the hypothesis that prenatal under‐ and overnutrition in late gestation can program small intestinal (SI) growth, angiogenesis, and endocrine function to predispose for a hyperabsorptive state, thereby increasing the susceptibility to the adverse effects of an early postnatal obesogenic diet. Twin‐pregnant ewes were exposed to adequate (NORM), LOW (50% of NORM), or HIGH (150% energy and 110% protein of NORM) diets through the last trimester (term ~147 days). From 3 days to 6 months of age, their lambs were fed either a moderate (CONV) or a high‐carbohydrate high‐fat (HCHF) diet. At 6 months of age, responses in plasma metabolites and insulin to refeeding after fasting were determined and then different segments of the SI were sampled at autopsy. Prenatal overnutrition impacts were most abundant in the duodenum where HIGH had increased villus amplification factor and lowered villi thickness with increased *IRS‐1* and reduced *GH‐R* expressions. In jejunum, HIGH lambs had an increased expression of *Lactate* gene and amplified when exposed to HCHF postnatally. Specifically, in LOW, sensitivity to HCHF was affected in ileum. Thus, the mismatching LOW‐HCHF nutrition increased expressions of angiogenic genes (*VEGF, VEGF‐R1, ANGPT1, RTK*) and increased mucosa layer (*tunica mucosa*) thickness but reduced muscle layer (*Tunica muscularis*) thickness. The SI is a target of prenatal nutritional programming, where late gestation overnutrition increased and shifted digestive capacity for carbohydrates toward the jejunum, whereas late gestation undernutrition predisposed for ileal angiogenesis and carbohydrate and fat hyperabsorptive capacity upon subsequent exposure to postnatal obesogenic diet.

## Graphical abstract

The small intestine is a target of prenatal nutritional programming, where late gestation overnutrition increased and shifted digestive capacity for carbohydrates toward the jejunum. Late gestation undernutrition predisposed for ileal angiogenesis and carbohydrate and fat hyperabsorptive capacity upon subsequent exposure to postnatal obesogenic diet.

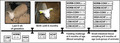

## 1 INTRODUCTION

Numerous studies in humans and different animal models have convincingly demonstrated that adverse prenatal nutrition exposures can impact metabolic and endocrine functions later in life (Barker et al., 1993; Boerschmann, Pflüger, Henneberger, Ziegler, & Hummel, 2010; Fabricius‐Bjerre et al., 2011; Howie, Sloboda, & Vickers, 2012; Meas et al., 2010; Samuelsson et al., 2008). This has been ascribed to the phenomenon termed fetal programming. Various key organs involved in nutrient metabolism, glucose–insulin axis function and development of adiposity have been studied to gain insight into the mechanisms, whereby adverse prenatal nutritional exposures are coupled to a predisposition for metabolic diseases, such as type 2 diabetes and obesity (de Freitas et al., 2003; Johnsen, Kongsted, & Nielsen, 2013; Khanal et al., 2014, 2020; Morris, Vickers, Gluckman, Gilmour, & Affara, 2009). The intestine has so far received limited attention in this regard. However, the role of the small intestine (SI) in relation to type 2 diabetes has been acknowledged over the last decades, after it was discovered that a specific type of obesity operation, called Roux‐en‐Y Gastric Bypass, results in complete and weight‐loss independent resolution of type 2 diabetes in obese individuals (Jiao et al., 2012; Kashyap et al., 2010; Pories et al., 1995; Thaler & Cummings, 2009). In the Roux‐en‐Y Gastric Bypass, the stomach is connected directly with the proximal jejunum, hence bypassing the duodenum, and the normalization of glucose–insulin axis function occurs within days or weeks after the operation. In good correspondence with this, studies of the SI and glucose absorption in diabetic rats have indicated that increased intestinal capacity for glucose absorption can be a contributing factor to diabetes (Adachi et al., 2003; Fujita et al., 1998). These findings made us speculate, whether the SI could also be a target of fetal programming and play a role in the predisposition for metabolic disorders such as type 2 diabetes later in life in individuals exposed to fetal malnutrition.

Small intestinal absorptive capacity is influenced by many factors, for example, number and activity of nutrient transporters (Debnam, Ebrahim, & Swaine, 1990; Fujita et al., 1998), but the first and foremost is the quantitative growth and development of the organ, including the mucosa (Adachi et al., 2003; Fujita et al., 1998; Zoubi, Mayhew, & Sparrow, 1995). The organogenesis of the intestine occurs quite early in gestation, but during the last 15 weeks of gestation in humans, the SI undergoes a remarkable longitudinal growth and maturation (Montgomery, Mulberg, & Grand, 1999; Pacha, 2000). During the early postnatal period (suckling and weaning), the intestinal mucosa undergoes further maturational changes in precocial species, which may be sensitive to dietary changes (Pacha, 2000). In the present study, we used a well‐documented precocial experimental model (the Copenhagen sheep model; Khanal et al., 2014) to test the hypothesis that prenatal under‐ and overnutrition in late gestation can program growth, angiogenesis, and endocrine function of the SI to predispose for a hyperabsorptive state, thereby increasing the susceptibility to the adverse effects of an early postnatal obesogenic high‐fat diet. The SI is not only an absorptive organ, but also an endocrine organ, that can affect whole body energy homeostasis by influencing appetite regulation and glucose homeostasis (Drucker, 2007; King, 2005). We therefore wished to include a focus on the incretin hormones: gastric inhibitory polypeptide (GIP) and glucagon‐like peptide 1 (GLP‐1), since their synthesis is stimulated by the presence of glucose and fatty acids in the small intestinal lumen, and they are implicated in blood glucose regulation by promoting insulin secretion (Drucker, 2007; Hirasawa et al., 2005; Reimann et al., 2008). Fetal programming of the SI incretin synthesis would have implications, in addition to SI development per se, for quantitative nutrient absorption, blood glucose levels, and hence the risk of development of both insulin resistance and obesity, which as mentioned above may have a fetal origin. This study will contribute with new knowledge on whether small intestinal functional development is differentially programmed by over‐ versus undernutrition in late gestation in a precocial animal model, and whether this may alter the susceptibility of small intestinal functional traits upon exposure to an early postnatal energy dense diet. Hence, it can be evaluated whether fetal programming of functional development of the small intestine may be involved in development of adverse metabolic outcomes previously observed in animals in this experiment.

## 2 MATERIALS AND METHODS

Animal experiments were conducted at the experimental facilities on the farm Rosenlund, Lynge, Denmark under the auspices of the Faculty of Health and Medical Sciences, University of Copenhagen, Frederiksberg, Denmark. All experimental procedures were approved by the National Committee on Animal Experimentation, Denmark.

### 2.1 Animals and experimental design

**FIGURE 1:**
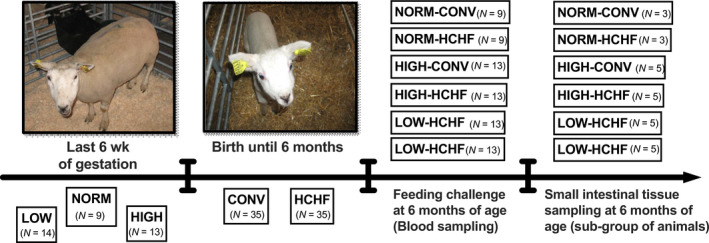
Overview of animal experiment and design. Late twin‐pregnant ewes (last 6 weeks of gestation) where fed either a NORM (100% of requirements for energy and protein in this stage of gestation, *N* = 9), a LOW (50% compared to NORM, *N* = 14), or a HIGH (150% of energy and 110% of protein requirements, *N* = 13) diet. After lambing their twin lambs were fed each their diet, either a conventional (CONV) diet (consisting of milk replacer during the first 8 weeks of life and green hay adjusted to achieve moderate growth rates of 225 g/day) or a high‐carbohydrate high‐fat (HCHF) diet (consisting of a milk/cream mix 1:1 supplemented with rolled maize) until 6 months of age. This 3 × 2 factorial design gave rise to six experimental groups, which were subjected to a fasting refeeding challenge at 6 months of age: NORM‐CONV (*N* = 9), NORM‐HCHF (*N* = 9), LOW‐CONV (*N* = 13), LOW‐HCHF (*N* = 13), HIGH‐CONV (*N* = 13), and HIGH‐HCHF (*N* = 13). Thereafter, subgroups of animals were killed and intestinal tissues samples were taken: NORM‐CONV (*N* = 3), NORM‐HCHF (*N* = 3), LOW‐CONV (*N* = 5), LOW‐HCHF (*N* = 5), HIGH‐CONV (*N* = 5), and HIGH‐HCHF (*N* = 5)

This study was part of a larger longitudinal study, and experimental animals and experimental design have been described in detail previously (Khanal et al., 2014). In brief, the experiment was a 3 × 2 factorial design, where 36 twin‐pregnant Texel ewes were allocated to one of three different dietary regimens during their third trimester, that is, the last 6 weeks of gestation (term ~147 days): a NORM (*N* = 9) diet fulfilling daily requirements for energy and protein (Danish feeding standards); LOW (*N* = 14) with a 50% reduction in dietary energy and protein compared to NORM; HIGH (*N* = 13) providing 150% of energy and 110% of protein requirements (Figure 1). After parturition, the twin offspring were allocated to one of two different diets fed from 3 days until 6 months of age: an obesogenic, high‐carbohydrate, high‐fat (HCHF) diet consisting of milk replacer mixed in a 1:1 (v/v) ratio with dairy cream (38% fat in dry matter) (max 2.5 L/d) and supplemented with rolled maize (max 2 kg/d); or a moderate conventional (CONV) diet consisting of milk replacer (until 8 weeks of age) and green hay administered in amounts to achieve moderate daily growth rates of 225 g/d. This gave rise to six experimental groups (two lambs died shortly after birth): NORM‐HCHF (*N* = 9), NORM‐CONV (*N* = 9). LOW‐HCHF (*N* = 13), LOW‐CONV (*N* = 13), HIGH‐HCHF (*N* = 13), and HIGH‐CONV (*N* = 13). A subgroup of animals were autopsied at 6 months of age as reported previously (Khanal et al., 2014, 2020): NORM‐HCHF (*N* = 3), NORM‐CONV (*N* = 3), LOW‐HCHF (*N* = 5), LOW‐CONV (*N* = 5), HIGH‐HCHF (*N* = 5), HIGH‐CONV (*N* = 5), and remaining animals continued in the experiment to be studied as adults (not reported here).

### 2.2 Blood samplings during refeeding after a 48‐hr fasting period and plasma analysis

At 6 months of age, lambs were equipped with an indwelling catheter in the jugular vein as described previously (Khanal et al., 2015) and subjected to 48 hr of fasting, where after they were refed with half of their daily ration. Blood samples were taken by the end of the fasting period just prior to refeeding (time point 0 relative to refeeding) and again 1 hr and 2½ hr after feeding. Samples were collected in EDTA‐coated tubes and placed on ice until centrifugation at 1,800*g*_av_ and 4°C for 15 min. Plasma was transferred to cryotubes and stored at −20°C.

Glucose, insulin, triglyceride, nonesterified fatty acids, cholesterol, lactate, β‐hydroxybutyrate, blood urea nitrogen, creatinine, and γ‐glutamyl transferase were measured in plasma samples according to methods previously described (Khanal et al., 2014).

### 2.3 Autopsy of SI at 6 months of age

A subgroup of 26 animals representing all six nutritional groups were selected for autopsy of the small intestine among other tissues at 6 months of age. Before killing, animals were anesthetized with propofol (5–6 mg i.m./kg body weight; B. Braun, Melsungen, Germany) and thereafter killed by debleeding by transection of both jugular veins and decapitation. The SI was excised immediately after the animals were killed and the individual regions of the SI (duodenum, jejunum, and ileum) were separated. The demarcation of duodenum proximally was the connection to the pylorus sphincter in the abomasum and distally as the last mesenteric vascularization which also drains the stomachs (also known as the ligament of Treitz). The ileum was identified proximally as the anatomical location, where the mesenteric blood circulation begins, which also drains the caecum, and distally as the entry into the large intestine. The jejunum was considered as the part of the small intestine lying in between these two regions, and lengths of all three regions were measured before sampling. From each region, tissue samples were collected 1/3 and 2/3 down into the region, to represent proximal (Duo1, Jej1, and Ile1) and distal parts (Duo2, Jej2, and Ile2) of the regions, respectively. Samples were gently cleaned by flushing with 0.9% NaCl solution before further processing.

### 2.4 RNA extraction, cDNA synthesis, and quantitative real‐time PCR (qPCR)

Tissue samples for mRNA gene expression analyses were immediately submerged in RNAlater (RNAlater^®^ Solution, Ambion, The RNA Company, USA) for 24 hr and then all samples were stored at −80°C until analyses. RNA extraction, analyses for concentration and integrity, as well as cDNA synthesis and qPCR were performed as previously described (Hou et al., 2013), except for the minor deviations stated below: About 50 mg of SI sample (a pool of approximately equal amounts taken from each of the two samples obtained from each region) was used for RNA extraction and the isopropanol phase separation step was performed at room temperature for 10 min. RNA integrity (RIN) was determined and a cutoff at 6.4 in RIN value was used. A quantity of 2 µg of extracted RNA was used in the reverse transcription PCR program of 25°C for 10 min → 42°C for 60 min → 95°C for 5 min → 4°C. A pool of produced cDNA from all samples and a 10× dilution of the pool (called calibrator) were made to be used as a base for standard curves and as an internal standard, respectively. The rest of the cDNA of each sample was then diluted 10 times to use as a working concentration during the qPCR (program: 45 cycles of 95°C for 10 s, 60°C for 10 s, at 72°C for 20 s). In the qPCR, beta‐actin was used as a reference gene and each primer pair was tested for specificity and the correct product. A melting curve of the PCR product was included in the program to ensure that only one single product was produced. Primer sequences and the origins of the primers are shown in Table 1. Primer pairs for *Lactase, Maltase, proGIP,* and *proGlucagon* were designed by PrimerDesign Ltd (Rownsham, Southampton, United Kingdom) and were run with the same procedure as mentioned above except for the use of a different program and reaction volume: 50 cycles of 95°C for 15 s and 60°C for 60 s with a reaction volume of 20 µl consisting of 5 µl of template, 1 µl of primer mix (incl. both forward and reverse primer), 10 µl of SYBR Green Mastermix, and 4 µl of PCR‐grade water. Two primer pairs (Glut‐5, Glc6Pase) had a supposed product above 500 bp and the program used was therefore slightly extended for these genes using 72°C for 20 s instead of 10 s. Primer product size was confirmed by gel electrophoresis and PCR products sequenced to confirm target.

**TABLE 1:** Primer sequences of the genes studied and the primer origin

Gene name	Forward primer	Reverse primer	Origin of primer pair
*Growth Hormone Receptor (GH‐R)*	GATCTCTGGCAGCTGCTGTT	GTGGCTTCACTCCCAGAAAAAG	(Husted, 2007)
*Insulin‐like Growth Factor 1 (IGF−1)*	TGTGATTTCTTGAAGCAGGTGAAG	GCTGAAGGCGAGCAAGCA	(Safayi et al., 2010)
*IGF−1 Receptor (IGF−1R)*	TGCAGAAGGAGCAGGTGACA	CCTCCACTTGGGATCCATATTTT	(Safayi et al., 2010)
*Insulin Receptor Substrate 1 (IRS−1)*	CACTTCGCCTACCATTTCC	TGCATTTCCAGACCCTCCT	(Hou et al., 2013)
*Vascular Endothelial Growth Factor (VEGF)*	GGGCTGCTGTAATGACGAAAG	TGAGGTTTGATCCGCATAATCTG	(Safayi et al., 2010)
*VEGF Receptor 1 (VEGF‐R1)*	TCAAGCCAATGTACAACAGGATGG	ATTAAACTTGGGAGCAGAAATATCTTCC	(Safayi et al., 2010)
*VEGF Receptor 2 (VEGF‐R2)*	CACTGTTTATGTGTATGTTCAAGATTAC	GATGTACACAACTTCATGCTGGTC	(Safayi et al., 2010)
*Angiopoetin 1 (ANGPT1)*	CCATAACCAGTCAGAGGCAGTAC	GTGTGACCCTTCAAATACAACCTG	(Safayi et al., 2010)
*Angiopoetin 2 (ANGPT2)*	CGAATGAAGAACTCAACTACAGGATTC	GAAGGACCACAGGCATCAAACC	(Safayi et al., 2010)
*ANGPT1 and 2 Receptor: tyrosine kinase tie2 receptor (RTK)*	AACTGTGACGACGAGGTGTATG	TCCCCGCGTAGGTGAACTTC	(Safayi et al., 2010)
*Sodium, Glucose Transporter 1 (SGLT1)*	GTTGGCTGTACCAACATCGCCTACC	CCAAGTAACTGGTGATGGACTGGAT	(Zhao, Okine, & Kennelly, 1999)
*Glucose Transporter 5 (Glut‐5)*	AGTCATCTCCATCATCGTCCT	GTACCCGCCACCATGTAGGCAG	(Rizos et al., 2004)
*Glucose‐6‐Phosphatase (Glc6Pase)*	GATAAAGCAGTTCCCGGTCA	ATCCAATGGCGAAACTGAAC	(Limesand, Rozance, Smith, & Hay Jr, 2007)
*Lactase*	GCTACCGCCTCATATCAGATC	GTCTCCTGTGTCATTGTTCTCA	PrimerDesign Ltd (copyright)
*Maltase*	CTGGGATGATGGGGAAACAAA	GGGGTCTGTGTAGGTTGATTG	PrimerDesign Ltd (copyright)
*Pro Gastric Inhibitory Peptide (ProGIP)*	AGTGACTACAGTATCGCCATG	CCTCTGGGTGATGTTGTGTAT	PrimerDesign Ltd (copyright)
*ProGlucagon*	CCGAGGAAGGCGAGATTTC	GGGTAGCAAGACTATCGAGAAC	PrimerDesign Ltd (copyright)
*Apolipoprotein A‐I (Apo A‐I)*	CTTGGCTGTGCTCTTCCTG	TCGAATTGGGCCACATAGTC	Designed by the second author
*Monoacylglycerol‐Acyltransferase 1 (MOGAT1)*	TGTCCCACGTGTTAAGCAAA	TGGCACCAAATAAGCACCAT	Designed by the second author
*Diacylglycerol‐Acyltransferase 1 (DGAT1)*	CACCATCCTCTTCCTCAAGC	AGTAGAGATCGCGGTAGGTC	Designed by the second author

### 2.5 Histological measurements and calculations

Immediately after sampling, the SI tissue samples were fixed in 4% paraformaldehyde (PFA (VWR, Herlev, Denmark) dissolved in PBS solution in MilliQ water (PBS tablets; Merck, Darmstadt, Germany) for approximately 24 hr and then kept in 1% PFA (for about a week) until embedding. The samples were then dehydrated overnight in a Jung TP 1050 tissue processor (Leica Microsystems A/S, Ballerup, Denmark) and afterwards embedded in paraffin. Two transverse sections of 5 µm thickness of SI were cut from each of the blocks on a microtome, placed on a glass slide, and stained with Mayer's hematoxylin (Merck, Darmstadt, Germany) and eosin (Merck, Darmstadt, Germany). The colored slides were mounted with DPX mounting media (VWR, Herlev, Denmark) and briefly examined in a light microscope. Good quality tissue slides were full slide scanned in a Panoramic MIDI Slide Scanner (3DHistech Kft., Budapest, Hungary). Most of jejunum tissues were of insufficient quality due to damage of villi, and this region could therefore not be included in these analyses.

#### 2.5.1 Surface areas

Pictures were taken of the full slide scan of the whole transection of SI segments in Panoramic viewer magnification 2.89× and then approximately 0.5 × 0.5 m sized printouts were made with magnification bars clearly indicated. As previously described (Mayhew, Middleton, & Ross, 1998), a boundary line was drawn around the whole printed transverse section at the base of villi and the opening of crypts, and the length of this boundary line (B(p); see below) represented the primary mucosa surface length. Then a grid consisting of equidistant vertical and horizontal lines (1 cm apart) was placed on top of the prints and covering the whole transection. This grid was used to count intersections between grid lines with surface of villi and with the primary mucosa boundary line. Finally, based on these measurements and the measured length of the SI regions, the total villus surface area (S(v)) in each of the sampled sites (1/3 and 2/3 down into the intestinal region) was derived at using the following sets of calculations (Mayhew et al., 1998):

$$S\left(v\right)=S_{s}\left(v,p\right)\times S\left(p\right)$$

where *S*(*p*) is the primary mucosa surface area and *S_s_*(*v*, *p*) is the villus amplification factor, which was calculated as:

$$S_{s}\left(v,p\right)=\frac{4}{\pi }\times \frac{I\left(v\right)}{I\left(p\right)}$$

where *I*(*v*) was determined as the total counts of intersections between grid lines and the villi surface, and *I*(*p*) was the total counts of intersections between the grid lines and the boundary line (primary mucosa surface). The quadratic grid took minor variations from circularity into account (Mayhew et al., 1998). The primary mucosa surface area was calculated as:

$$S\left(p\right)=L\times B\left(p\right)$$

where *L* was the length of the intestinal region and *B*(*p*) was the length of the boundary line, which in turn was derived from *I*(*p*) (total counts of intersections between the grid lines and the boundary line (primary surface)) and the dimensional spacing (h) of the lines in the grid (1 × 1 cm with magnification 2.89×) as follows:

$$B\left(p\right)=\frac{\pi }{4}\times h\times I\left(p\right)$$

#### 2.5.2 Layers

Another set of pictures were taken specifically of the mucosa (*tunica mucosa;* 7–8 pictures/slide) and the outer muscle layers (*tunica muscularis;* 3–8 pictures/slide; only in 14.8% of slides were numbers of pictures taken ≤5) in the scanned slides using a Panoramic Viewer at 8× magnification. Using a randomly translated point grid, two villi per mucosa picture were selected. Mucosa layer (*tunica mucosa*) thickness was measured as the distance between the apical point of each villus to the closest point in the muscularis mucosae layer (*lamina muscularis mucosa*) beneath. At the middle height of each villus, its width (from apical membrane to apical membrane of epithelial cells), its middle layer (*lamina propria*) thickness (from basal membrane to basal membrane of epithelial cells), and villus epithelial layer thickness (from apical to basal membrane of epithelial cell) were also measured. In the muscle layer (*tunica muscularis*) pictures, the thickness of both inner circular (*stratum circulare*) and outer longitudinal (*stratum longitudinale*) layers was measured at two points per picture using a randomly translated point grid. Measurements were performed using pixels and afterwards translated into µm using the conversion 1 pixel = 1.5384615 µm. The illustration of a slide picture showing mucosa (*tunica mucosa*) and muscle (*tunica muscularis*) layers with different structures, and the marking and labelling of specific measurements performed in the present study are provided in the Figure S1.

### 2.6 Statistics

A generalized linear model with or without repeated measurements, depending on whether the regions were analyzed together (histology) or separately (gene expressions and small intestinal lengths), was used with lamb nested within sheep as random effects. The feeding challenge data were analyzed using a model with repeated measurements (time points during the challenge) with lambs and sheep as random effects, since all animals (twin pairs) were challenged. Ewe body weight and body condition score at the start of the experiment (6 weeks prepartum) were used to assign ewes to their treatment groups, and sex (first priority) and birth weight (second priority) of newborn lambs were used to assign twin lambs to each their postnatal treatment groups. This was to ensure as even a distribution as possible of sex and birth weight across treatment groups. Lamb body weight at 3 months of age was used for selection of the lambs to be killed. The full models for the feeding challenge data therefore included ewe start body weight and body condition score, lamb sex, and birth weight as covariates and the full models for the qPCR data included in addition to these also the lamb body weight at 3 months as covariate. At last, stepwise reduction of the models was performed removing insignificant factors from the models. All data are presented as Least Square Means (LSM) with 95% confidence limits (Cl), except for data for feeding challenge, which are presented as LSM with Standard Error of Means (*SEM*).

## 3 RESULTS

From the same experimental study, we have previously published results regarding long‐term impacts of early life nutrition on growth trajectory and various aspects of metabolic and endocrine adaptability (Khanal et al., 2014, 2015). Selected results from these previous publications will be included in the discussion, when it was deemed relevant for interpretation of data relating to nutritional programming of the small intestine from the present study. In the following, no effects were detected of prenatal nutrition, postnatal nutrition, or sex unless explicitly stated.

### 3.1 Plasma metabolite and insulin profiles in response to refeeding after fasting

**FIGURE 2:**
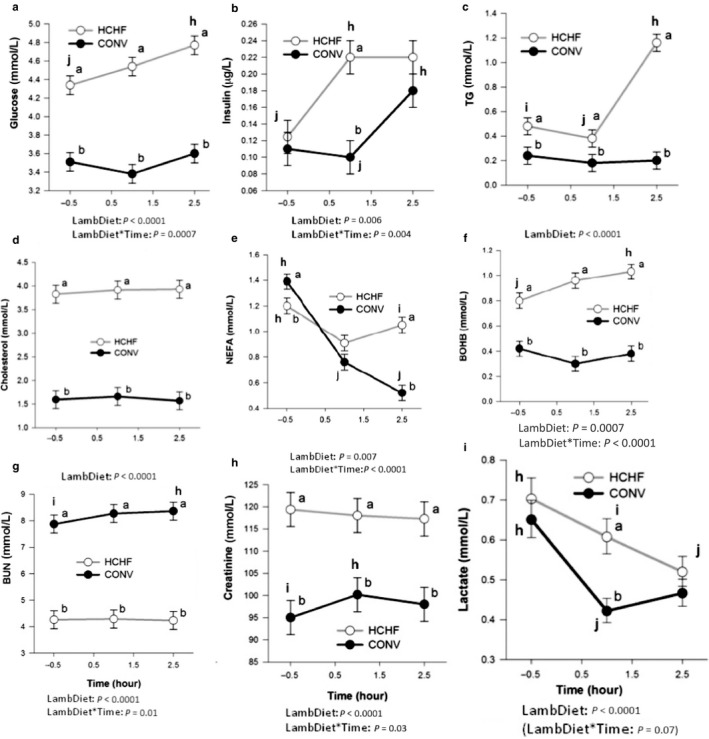
Impacts of postnatal nutrition on plasma metabolite profiles during refeeding after a 48‐hr fasting period. (a) Glucose, (b) insulin, (c) TG (triglycerides), (d) cholesterol, (e) NEFA (nonesterified fatty acids), (f) BOHB (β‐hydroxybutyrate), (g) BUN (blood urea nitrogen), (h) creatinine, and (i) lactate. (a and b) Significantly different between the postnatal dietary groups (post hoc test): (h, i, j) Significantly different between the time points. Lamb Diet: postnatal dietary treatments. For HCHF, CONV, see legends to Figure 1. Data are reported as least square (LS) means ± *SEM* shown as vertical bars

Clear effects of the postnatal diet were observed on all blood parameters measured before and after refeeding after a 48‐hr fasting period ( Figure 2), whereas no effects of the prenatal diet were observed. After 48 hr of fasting, the HCHF compared to CONV lambs had markedly higher plasma levels of glucose (4.34 vs. 3.51 mM, respectively; Figure 2a), triglyceride (0.48 vs. 0.24 mM, respectively; Figure 2c), cholesterol (3.83 vs. 1.59 mM, respectively; Figure 2d), β‐hydroxybutyrate (0.80 vs. 0.42 mM, respectively; Figure 2f), creatinine (119.4 vs. 95.0 μM, respectively; Figure 2h), and γ‐glutamyl transferase (155.4 vs. 59.0 U/l, respectively; Figure S2), while fasting plasma levels were lowest in HCHF for nonesterified fatty acids (1.20 vs. 1.39 mM, respectively; Figure 2e) and blood urea nitrogen (4.26 vs. 7.87 mM, respectively; Figure 2g).

Upon refeeding, glucose and β‐hydroxybutyrate plasma levels remained low and relatively unaffected in CONV, but increased steadily over the following 2.5 hr in HCHF lambs (*p* = .0007 and *p* < .0001 for the lamb diet and time interaction, respectively), whereas the reverse picture was observed for blood urea nitrogen (*p = *.01 for the lamb diet and time interaction). Insulin concentrations increased in all lambs after refeeding, but most pronounced and most rapidly in HCHF (*p* = .004 for lamb diet and time interaction). This was associated with a marked depression in plasma nonesterified fatty acid levels in CONV lambs to approach normal values 2.5 hr after refeeding (*p* < .0001 for lamb diet and time interaction). Nonesterified fatty acid levels were also decreased one hour after refeeding in the HCHF lambs, but increased thereafter to close to fasting levels, during which time plasma triglyceride levels had more than doubled compared to fasting levels (increased from 0.48 to 1.16 mM, *p* < .0001 for lamb diet and time interaction). Triglyceride levels were not affected by refeeding in the CONV lambs. The 48‐hr fasting lactate levels were similar in CONV and HCHF lambs, and lactate levels decreased after refeeding to the same lower levels in all groups, but the decrease occurred most rapidly in the CONV group (*p* < .0001 for time). Plasma levels of the other measured parameters did not change after refeeding (cholesterol) or changes were quantitatively insignificant (creatinine *p* = .03, γ‐glutamyl transferase *p* = .009).

### 3.2 Small intestinal (SI) development and histological features

Histological evaluations were, as previously mentioned, conducted only on duodenal and ileal samples due to lack of quality of jejunal samples.

#### 3.2.1 Prenatal impacts

**FIGURE 3:**
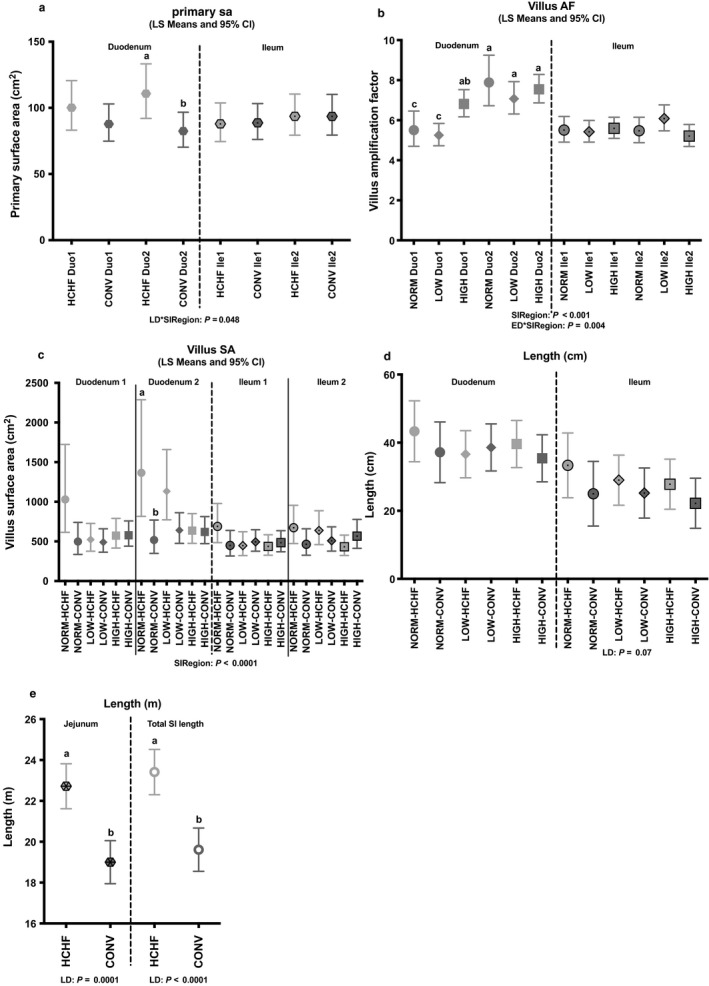
Impacts of pre‐ and postnatal nutrition on small intestinal surface area (duodenum and ileum) and length (jejunum and total length shown). (a) Primary surface area, (b) villus AF (amplification factor), (c) villus surface area, (d) length (duodenum and ileum), and (e) Length (jejunum and total small intestinal length). Values are displayed according to any overall significant effects of pre‐ or postnatal nutrition and small intestine region (not tested in length as they were analyzed separately) as well as the interactions as detected in the fixed effects test (see *p*‐values below regions). (a–c) Significantly different between treatment groups (post hoc test) within the intestinal regions. SA, surface area; AF, amplification factor; ED, ewe diet (prenatal nutrition); LD, lamb diet; SIRegion, small intestinal region; Duo1, duodenum region 1; Duo 2, duodenum region 2; Ile 1, ileum region 1; Ile 2, ileum region 2; SI, small intestine. For NORM, LOW, HIGH, CONV, and HCHF, see legends to Figure 1. Data are reported as least square (LS) means with 95% confidence limits (Cl) as vertical bars

**FIGURE 4:**
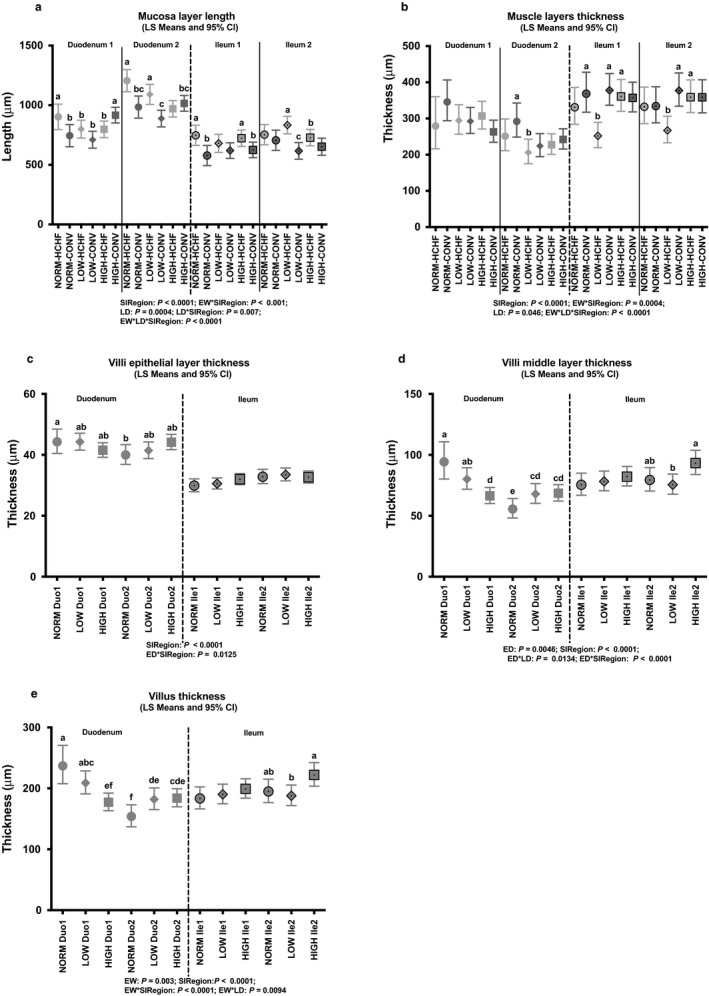
Impacts of pre‐ and postnatal nutrition on mucosa and villi thicknesses in the small intestine (duodenum and ileum). (a) Mucosa layer length, (b) muscle layer thickness, (c) villi epithelial layer thickness, (d) villi middle layer thickness, and (e) villus thickness. Values are displayed according to any overall significant effects of pre‐ or postnatal nutrition and small intestine region as well as the interactions as detected in the fixed effects test (see *p*‐values below regions). (a–f) Significantly different between treatment groups (post hoc test) within intestinal regions. For NORM, LOW, HIGH, CONV, and HCHF, see legends to Figure 1. For ED, LD, SIRegion, Duo1, Duo2, Ile1, Ile2, and SI, see legends to Figure 3. Data are reported as least square (LS) means with 95% confidence limits (Cl) as vertical bars

As shown in Figures 3 and 4, the impacts of prenatal nutrition on SI morphology were predominantly observed in the duodenum, but with differences between the proximal (Duo1) and distal (Duo2) parts. Hence, villus amplification factor was increased in HIGH compared to NORM and LOW in Duo1, with no differences observed in Duo2 (Figure 3b). In Duo1, villi and villi middle layer (*lamina propria*) were thinnest in HIGH and thickest in NORM, but in Duo2 they were thinnest in NORM and thickest in LOW and HIGH (Figure 4d and e). The only impact of prenatal (independently of postnatal) nutrition in ileum was observed in the distal part (Ile2), where villi were thickest in HIGH and thinnest in LOW with NORM in between (Figure 4e). All other parameters were unaffected by the prenatal nutrition.

#### 3.2.2 Postnatal impacts

The total SI length was increased in HCHF compared to CONV lambs, predominantly due to an increased jejunal length (*p < *.0001; Figure 3e).

#### 3.2.3 Pre‐ and postnatal interactions

In duodenum (both Duo1 and Duo2), NORM and LOW lambs that had been fed the HCHF as compared to CONV diet had increased mucosa layer (*tunica mucosa*) thickness, whereas HIGH lambs had the opposite response to the HCHF diet (Figure 4a). In the ileum (both Ile1 and Ile2), LOW lambs exposed to the HCHF diet had the thinnest muscle layer (*tunica muscularis*) compared to other groups (Figure 4b).

### 3.3 Gene expression of endocrine signaling markers

#### 3.3.1 Prenatal impacts

**FIGURE 5:**
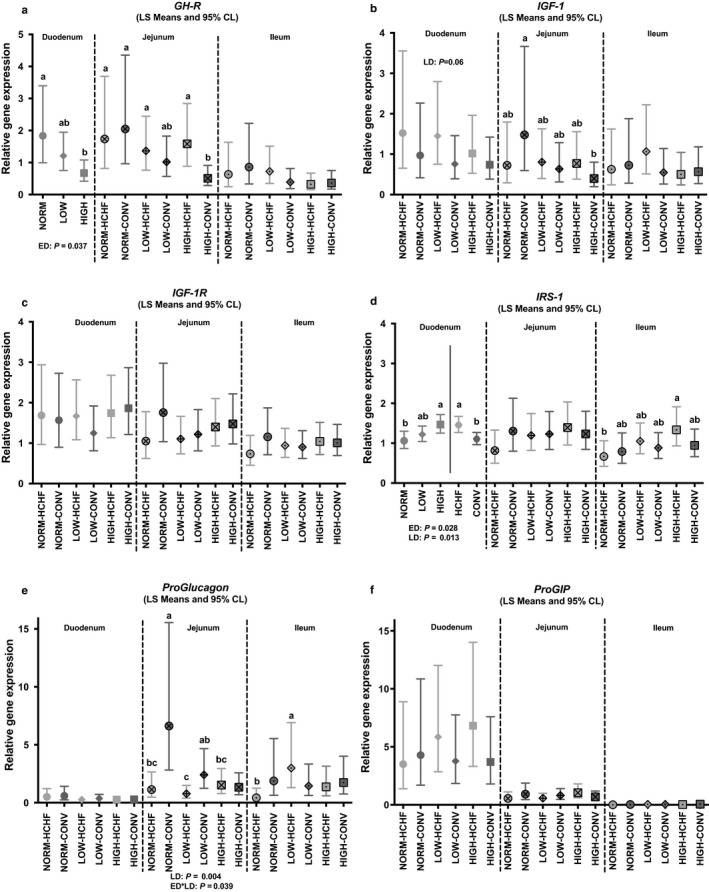
Impacts of pre‐ and postnatal nutrition on mRNA expression of genes involved in growth hormones and endocrine/incretin effects in the small intestine (duodenum, jejunum, and ileum). (a) *GH‐R* (growth hormone receptor), (b) *IGF‐1* (insulin‐like growth factor 1), (c) *IGF‐1R* (insulin‐like growth factor 1 receptor), (d) *IRS‐1* (insulin receptor substrate 1); (e) *ProGlucagon*, and F. *ProGIP* (pro‐gastric inhibitory peptide). Values are displayed according to any overall significant effects of pre‐ or postnatal nutrition as well as their interactions as detected in the fixed effects test (see *p*‐values below regions) within intestinal regions. (a–c) Significantly different between treatment groups (post hoc test) within intestinal regions. For NORM, LOW, HIGH, CONV, and HCHF, see legends to Figure 1. For ED and LD, see legends to Figure 3. Data are reported as least square (LS) means with 95% confidence limits (Cl, vertical bars) and represent normalized expression values according to the internal standard (calibrator) and relative to the beta‐actin reference gene expression

The expressions of *GH‐R* (*p = *.037; Figure 5a) and *IRS‐1* (*p = *.028; Figure 5d) genes were significantly affected by prenatal nutrition in the duodenum. The highest expression level of *GH‐R* gene was observed in NORM followed by LOW and lowest in HIGH (significantly lower than NORM), whereas *IRS‐1* gene expression was highest in HIGH followed by LOW and then NORM (significantly lower than HIGH).

#### 3.3.2 Postnatal impacts

Impacts of postnatal diet alone were observed in the duodenum, where HCHF fed lambs had increased the expressions of *IGF‐1* (*p = *.06; Figure 5b) and *IRS‐1* (*p = *.013; Figure 5d) genes compared to CONV fed lambs.

#### 3.3.3 Pre‐ and postnatal interactions

A significant interactive effect of pre‐ and postnatal nutrition was observed for *ProGlucagon* gene (*p = *.039; Figure 7e) in the jejunum, where the HCHF diet depressed expression in NORM and LOW, but not in HIGH lambs. Hence, NORM‐CONV lambs had the highest expression and the lowest expressions were found in the HIGH‐CONV and HIGH‐HCHF lambs.

### 3.4 Gene expression of angiogenesis markers

#### 3.4.1 Postnatal impacts

**FIGURE 6:**
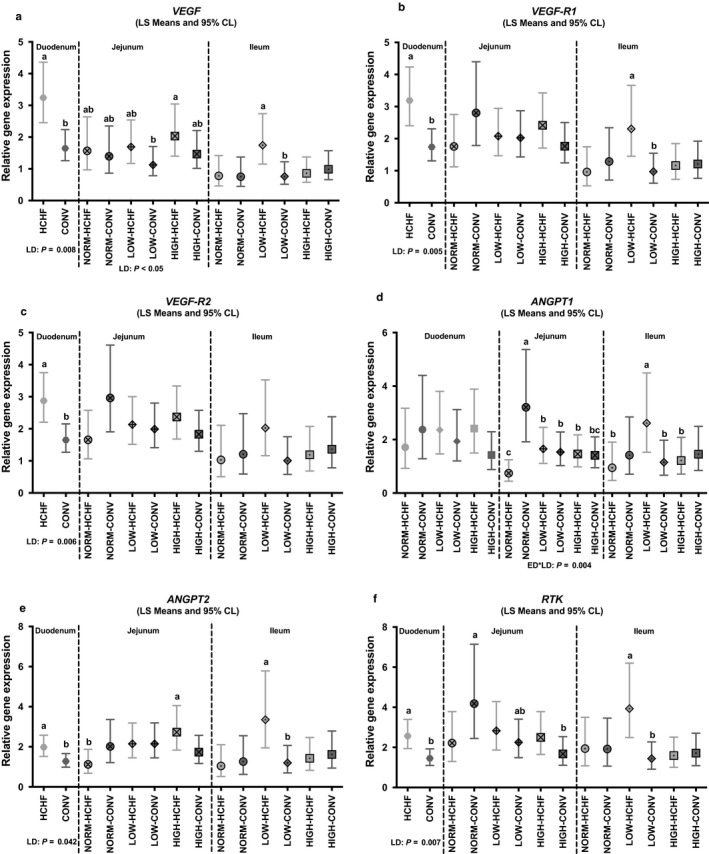
Impacts of pre‐ and postnatal nutrition on mRNA expression of genes involved in angiogenesis in the small intestine (duodenum, jejunum, and ileum). (a) *VEGF* (vascular endothelial growth factor), (b) *VEGF‐R1* (vascular endothelial growth factor receptor 1), (c) *VEGF‐R2* (vascular endothelial growth factor receptor), (d) *ANGPT1* (Angiopoetin 1), (e) *ANGPT2* (Angiopoetin 2), (f) *RTK* (*ANGPT1* and 2 receptor: tyrosine kinase tie2 receptor). (a–c) Significantly different between treatment groups (post hoc test) within intestinal regions. For NORM, LOW, HIGH, CONV and HCHF, see legends to Figure 1. For ED and LD, see legends to Figure 3. Data are reported as least square (LS) means with 95% confidence limits (Cl, vertical bars) and represent normalized expression values according to the internal standard (calibrator) and relative to the beta‐actin reference gene expression

In the duodenum, HCHF lambs had increased expressions of *VEGF* (*p = *.008; Figure 6a), *VEGF‐R1* (*p = *.005; Figure 6b), *VEGF‐R2* (*p = *.006; Figure 6c), *ANGPT2* (*p = *.042; Figure 6e) and *RTK* (*p = *.007; Figure 6f) genes compared to CONV lambs.

#### 3.4.2 Pre‐ and postnatal interactions

In the jejunum, NORM‐CONV and NORM‐HCHF lambs had the highest and lowest, respectively, of ANGPT1 (*p* = .004; Figure 6d) (similar tendency also for *RTK*; Figure 6f) with other groups in between. For ANGPT2, jejunal expression was highest in HIGH‐HCHF lambs (significantly higher than NORM‐HCHF; Figure 6e). The LOW‐HCHF lambs had significantly higher ileal expressions than the LOW‐CONV for several genes (significant in *ANGPT1*, *ANGPT2* and *RTK*, tendency observed for *VEGF‐R2*; Figure 6).

### 3.5 Gene expression of carbohydrate and lipid absorption markers

#### 3.5.1 Prenatal impacts

**FIGURE 7:**
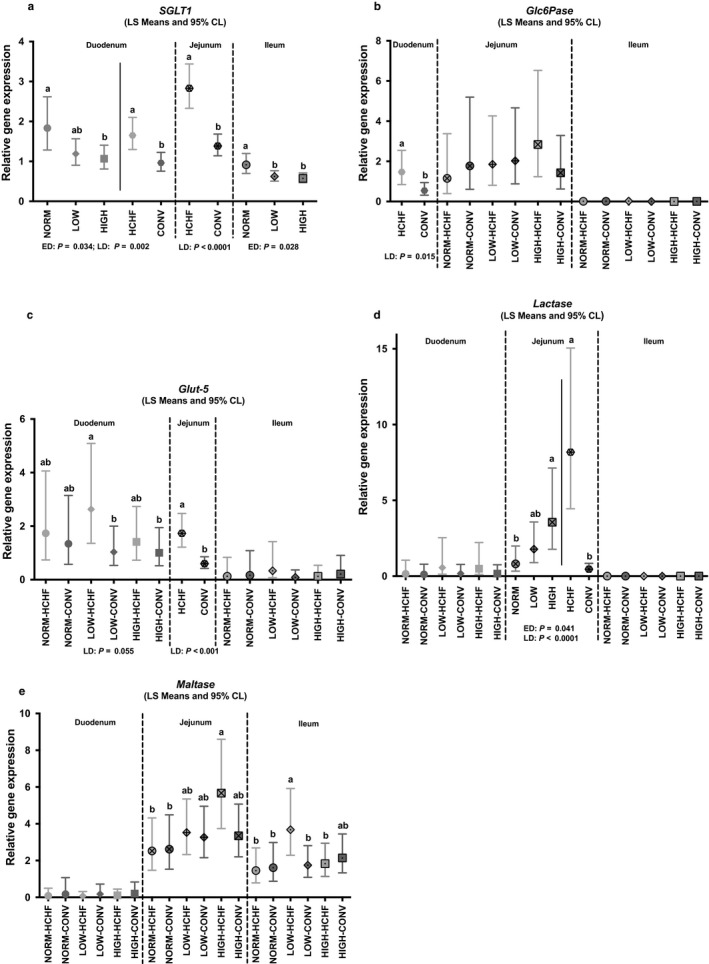
Impacts of pre‐ and postnatal nutrition on mRNA expression of genes involved in carbohydrate absorption in the small intestine (duodenum, jejunum and ileum). (a) *SGLT1* (sodium‐glucose transporter 1), (b) *Glc6Pase* (glucose‐6‐phosphatase), (c) *Glut‐5* (glucose transporter 5, facultative fructose transporter), (d) *Lactase*, and (e) *Maltase*. (a–c) Significantly different between treatment groups (post hoc test) within intestinal regions. For NORM, LOW, HIGH, CONV, and HCHF, see legends to Figure 1. For ED and LD, see legends to Figure 3. Data are reported as least square (LS) means with 95% confidence limits (Cl, vertical bars) and represent normalized expression values according to the internal standard (calibrator) and relative to the beta‐actin reference gene expression

In both duodenum (*p = *.034) and ileum (*p = *.023), the *SGLT1* gene was highest expressed in NORM and lowest in HIGH lambs (significantly lower than NORM; Figure 7a). In jejunum, the *Lactate* gene had the highest expression in HIGH (*p = *.04 relative to NORM) and lowest in NORM lambs (Figure 7d).

#### 3.5.2 Postnatal impacts

**FIGURE 8:**
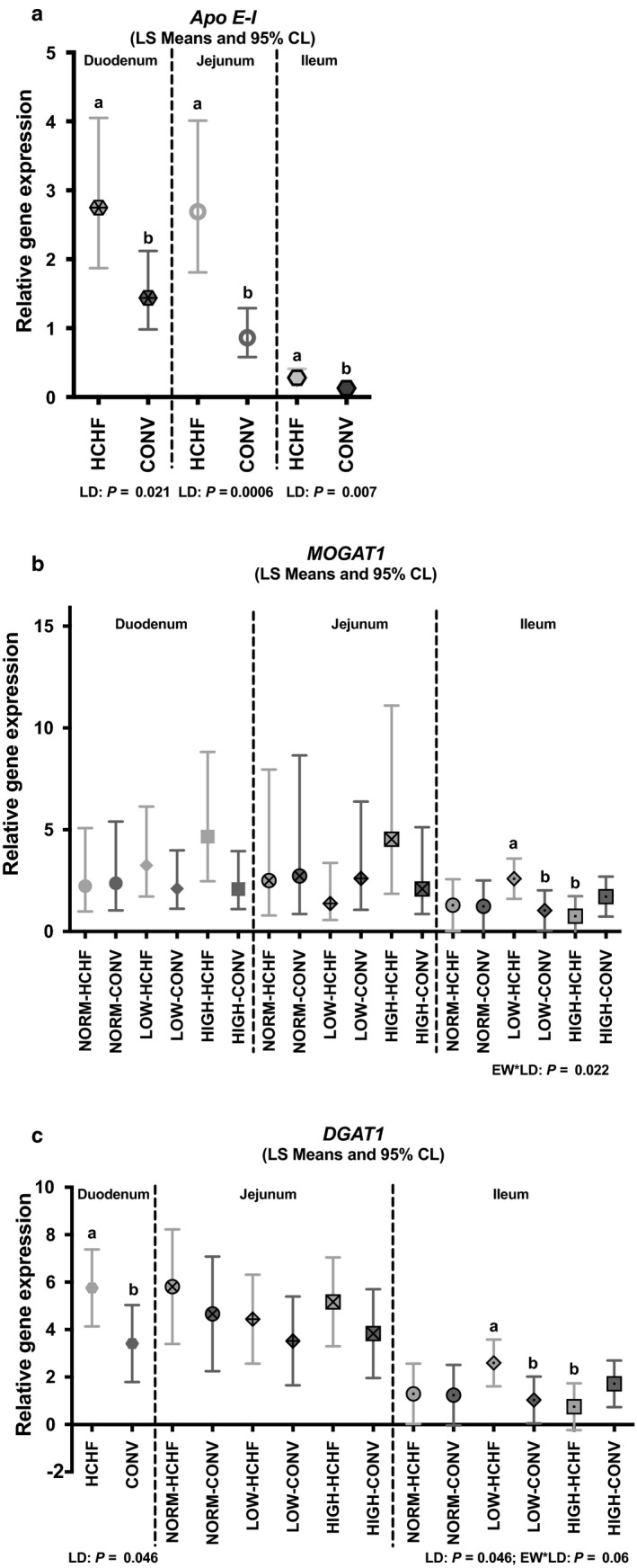
Impacts of pre‐ and postnatal impacts on mRNA expression of genes involved in fat absorption in the small intestine (duodenum, jejunum, and ileum). (a) *Apo A‐I* (apolipoprotein A‐I), (b) *MOGAT1* (monoacylglycerol‐acyltransferase 1), and (c) *DGAT1* (diacylglycerol‐acyltransferase 1). (a and b) Significantly different between treatment groups (post hoc test) within intestinal regions. For NORM, LOW, HIGH, CONV, and HCHF, see legends to Figure 1. For ED and LD, see legends to Figure 3. Data are reported as least square (LS) means with 95% confidence limits (Cl, vertical bars) and represent normalized expression values according to the internal standard (calibrator) and relative to the beta‐actin reference gene expression

In the duodenum, the HCHF fed lambs had increased gene expressions of *SGLT1* (*p* = .002; Figure 7a), *Glc6Pase* (*p* = .015; Figure 7b), *Glut 5* (*p* = .0546; mainly in LOW lambs; Figure 7c), and *DGAT1* (*p = *.046; Figure 8c) compared to CONV fed lambs. In the jejunum, the HCHF diet increased gene expressions of *SGLT1* (*p* < .0001), *Glut 5* (*p* = .0002) and *Lactase* (*p* < .0001: Figure 7d). The expression of *Apo E‐I* gene (Figure 8a) was increased in HCHF compared to CONV fed lambs in both duodenum (*p = *.021), jejunum (*p = *.0006) and ileum (*p = *.07).

#### 3.5.3 Pre‐ and postnatal interactions

The LOW‐HCHF lambs had the highest (significantly higher than LOW‐CONV and HIGH‐HCHF lambs) expressions of both *MOGAT1* (*p = *.022; Figure 8a) and *DGAT1* (*p = *.06; Figure 8c) genes in the ileum.

## 4 DISCUSSION

Using a precocial experimental animal model, namely sheep, we report here one of a very limited number of studies regarding impacts of late gestation malnutrition on growth, absorptive capacity, and endocrine signaling of the SI. Our study in addition addresses the implications this fetal programming may have for adaptation to widely different diets in early postnatal life. Although we used a ruminant animal as an experimental model, the HCHF diet was designed in such a way (see Nielsen et al., 2013) that a significant proportion of carbohydrate (starch and lactose) and fat (dairy cream) digestion took place in the small intestine. Maize starch has a low degradability in the rumen allowing it to escape rumen fermentation, and when young ruminants suckle, an esophageal groove reflex causes muscle flaps to form a tube that directs the ingested liquid (containing lactose and milk fat) directly to the lower segments of the gut, thereby bypassing forestomach fermentation. Thus, the postprandial changes in plasma metabolites we observed in the present study confirmed that postprandial patterns of nutrient absorption in HCHF lambs resembled that of monogastric animals.

### 4.1 Prenatal nutrition and SI digestive and absorptive capacity

Within the last decade, evidence has been presented to suggest that the functional development of the SI may be affected by nutrition in prenatal life. Hence, it has been shown in rats that a diet high in prebiotic fibers (lowered energy density) fed to dams throughout pregnancy and lactation increased the expression of both *SGLT1* and Glut‐5 in the SI of 28 days old offspring (Maurer & Reimer, 2011). Moreover, maternal low protein supply increased the mRNA and protein expressions of nutrient‐responsive receptors and transporters such as taste receptor type 1 member 3 (*T1R3*), *SGLT1,* and *GLUT2* in the intestine of offspring (Wang et al., 2020). In addition, high fiber or high‐fat diets fed to pregnant dams also led to increased intestinal weight or length in the offspring (Fak, Karlsson, Ahrne, Molin, & Westrom, 2012; Maurer & Reimer, 2011). Thus lowered nutrient/energy supply in prenatal life may increase the capacity for glucose absorption in postnatal life, and this could be a factor contributing to explain why prenatally malnourished individuals have an increased risk later in life for development of type 2 diabetes (Boerschmann et al., 2010; Fabricius‐Bjerre et al., 2011; Samuelsson et al., 2008; Stein, Obrutu, Behere, & Yajnik, 2019).

Nutritional interventions during pregnancy in altricial animal models as the rat do not target a period of fetal development equivalent to the third trimester in precocial animals as already mentioned. Our study showed that third trimester malnutrition had long‐term implications for proximal SI development, but prenatal over‐ and undernutrition had differential impacts. In HIGH lambs, which were subjected to prenatal overnutrition, the villus amplification factor was increased in the proximal part of duodenum, suggesting a higher surface area for absorption in this region. This may be related to an increased duodenal expression of the IRS‐1 gene. Insulin has been shown to be an important growth factor in the SI (Ménard, Corriveau, & Beaulieu, 1999; Sukhotnik et al., 2005), and *IRS‐1* is involved in insulin signaling (Marandi et al., 2001). Nevertheless, HIGH lambs had the lowest duodenal and ileal expressions of *SGLT1*, so the overall absorptive capacity for glucose may not have been increased in neither duodenum nor ileum. However, the jejunum is quantitatively the most important part of the SI in relation to carbohydrate digestion, due to its length and far more extensively developed surface area (DeSesso & Jacobson, 2001). HIGH lambs had increased expressions of lactase in the jejunum, and also of maltase upon exposure to the HCHF diet postnatally, which points to increased capacity for carbohydrate digestion and absorption in this part of the SI. This is supported by previously reported observations from the same animals (Khanal et al., 2014), where the hyperglycemic effect of the postnatal obesogenic HCHF diet was found to be more pronounced in HIGH compared to LOW and NORM lambs. Thus, prenatal overnutrition in the last trimester of fetal development may increase the absorptive and digestive capacity for carbohydrates specifically in the jejunum, thus predisposing for hyperglycemia upon exposure to high carbohydrate diets in postnatal life. It is not possible to evaluate to what extent changes in insulin (increased *IRS‐1* expression) or GH (decreased GHR expression) signaling in the duodenum may be part of an underlying mechanism.

Many of the changes observed in HIGH lambs in the duodenum were also observed in the prenatally undernourished LOW lambs, although less pronounced. However, distinct changes compared to all other groups were observed in the ileum in LOW lambs that had been fed the mismatching HCHF diet in postnatal life. Thus, the LOW‐HCHF group had increased expression of all studied angiogenic genes (significantly or numerically), increased expression of genes involved in starch (maltase), and lipid (*MOGAT1* and *DGAT1*) digestion, as well as thinner villi and reduced muscle layer (*tunica muscularis*) thickness compared to all other groups. Both the maternal LOW and the postnatal HCHF diets contained low amounts of protein, which may explain the reduced ileal muscle layer (*tunica muscularis*) thickness, although we did not see an overall decrease in body muscle mass of this group to indicate that body protein synthesis as such was negatively affected in this group (Khanal et al., 2014). However, the intestine is especially sensitive to nutrient absorption, and it has been reported that tissue (muscle) atrophy in this organ in response to protein malnutrition can be disproportionate to the degree of changes occurring in other tissues (Ferraris & Carey, 2000; Tappenden, 2006). The observed gene expression changes in the LOW‐HCHF lambs clearly suggest an increased digestive and absorptive capacity of the ileum due to increased angiogenesis, and hence vascularity, as well as increased formation of enzymes involved in starch and lipid digestion. The decreased ileal muscle mass could also indirectly contribute to a more efficient digestion, due to reduced diffusion distances for absorbed nutrients, less efficient peristaltic movements, and hence longer retention times in the ileum (DeSesso & Jacobson, 2001).

As previously reported, the LOW‐HCHF lambs developed hypercholesterolemia, hyperuremia, and hypercreatinemia after they had been exposed to the HCHF diet for 6 months (Khanal et al., 2015), and these undesirable metabolic traits persisted into adulthood, even after they had been converted to the normal low‐fat CONV diet for 2 years (Khanal et al., 2016). This gives rise to the intriguing question, whether fetal programming of absorptive capacity of the ileum in response to late gestation undernutrition could play a particular role in predisposing for hypercholesterolemia and the metabolic syndrome upon exposure to a high‐fat diet later in life?

In any case, the many changes, although sometimes small, in gene expression and developmental characteristics point to the SI as an important target organ for late gestation nutritional programming, but with differential impacts of late gestation over‐versus undernutrition, where particularly prenatally undernourished individuals have adaptive disadvantages to high‐fat diets in postnatal life.

### 4.2 Prenatal nutrition and the SI endocrine system

The SI synthesizes the peptide hormone precursor, ProGlucagon, which posttranscriptionally is hydrolyzed to produce the hormone *GLP‐1*, which is secreted in response to presence of glucose and fatty acids in the SI lumen (Drucker, 2007; Song et al., 2019). *GLP‐1* is an incretin hormone that stimulates insulin secretion (Nauck & Meier, 2018; Salehi, Prigeon, & D’Alessio, 2011) even in fasting situations (Holst, 2007). The HIGH‐HCHF lambs in this study became hyperglycemic (Khanal et al., 2014) compared to all other groups, and we hypothesized that a blunting of their incretin hormone synthesis in response to ingestion of glucose and fat could be an underlying mechanisms explaining this dysregulation of glucose homeostasis. However, we did not see any prenatal effects in this study on postprandial changes in glucose and insulin, when the lambs were refed after a 48‐hr fasting period, and we did not see any convincing differences in Proglucagon expression in any parts of the SI to support this speculation. When ProGlucagon posttranscriptionally is cleaved in the SI, other hormones are also formed, like the intestinotrophic *GLP‐2* (Holst, 2007). Since *GLP‐1* or *GLP‐2* assays validated for sheep to our knowledge do not exist, we are unable to say whether changes in *GLP‐1* or *‐2* may have occurred even in the absence of changes in Proglucagon.

Likewise, out study did not suggest that small intestinal regulation of glucose‐insulin regulation via gastric inhibitory peptide (GIP) or intestinal insulin or GH‐IGF‐1 signaling could account for the differential changes observed in intestinal development and gene expression patterns in prenatally over‐ and undernourished lambs in this study. Future studies are therefore needed to unravel the underlying mechanisms.

### 4.3 A postnatal obesogenic diet had major impact on SI longitudinal growth and absorptive capacity

High‐fat diets in both rats (de Wit et al., 2008) and our study with lambs were associated with a massive longitudinal growth of the SI. In our study, this could be ascribed to growth of particularly the jejunum, which increased by an impressive ~4 m in HCHF compared to CONV lambs, although small increases in lengths of duodenum and ileum were also observed. We are unfortunately unable to say, whether this was associated with changes in the jejunal villi and mucosa structures, since it was not possible to conduct histological analyzes on jejunal tissues, due to extensive damage to villi in the collected samples. However, primary surface area in the last part of duodenum and mucosa layer (villi) length in both duodenum and ileum suggests that the HCHF diet results in a dramatic overall increase in SI development and absorptive capacity. This is in line with the marked (for a ruminant animal) postprandial increases in absorption of glucose and TG, which we observed upon refeeding the HCHF diet after a period of fasting, and as previously reported (Khanal et al., 2014) HCHF fed lambs had generally increased plasma levels of glucose absorption, TG and leptin similar to findings in growing rats fed high‐fat diets (de Wit et al., 2008; McAllan et al. 2013).

Nutrient occurs predominantly in the duodenum and proximal part of the jejunum (DeSesso & Jacobson, 2001), and the HCHF diet specifically increased the expression of all angiogenic genes (except *ANGPT1*) in the duodenum, as well as the genes involved in monosaccharide absorption (*SGLT1*, *Gl6Pase* except in jejunum, *GLUT5*), carbohydrate digestion (lactase), and lipid digestion (*Apo A‐I, DGAT1*) in both the duodenum and jejunum, but not ileum (except for *ApoA1*).

Growth and development of the small intestinal vascular system is obviously important for efficiency of nutrient absorption (Chou & Coatney, 1994; Martin et al., 2009; Pappenheimer & Michel, 2003), and angiogenic factors, such as *VEGF* and *ANGPT*, contribute significantly to the angiogenesis process in the SI (Conway, Collen, & Carmeliet, 2001; Schlieve et al., 2016). The observed increase in gene expression of lactase, *SGLT1*, *Glut‐5*, *Glc6Pase*, *Apo A‐I,* and *DGAT1* in one or more of the three SI regions shows that luminal nutrients can regulate the abundance or activity of epithelial proteins involved in carbohydrate and lipid digestion and absorption (Abumrad, Nassir, & Marcus, 2016; Black & Rohwer‐Nutter, 1991; Dyer, Hosie, & Shirazi‐Beechey, 1997; Shirazi‐Beechey et al., 1991).

Such a massive increase in digestive and absorptive capacity upon exposure to an obesogenic diet in young individuals obviously represents a significant risk factor in relation to development of metabolic syndrome, obesity, and related disorders (Kongsted et al., 2014; Panchal et al., 2011).

The upregulation of markers for angiogenesis by the HCHF diet could not be related to changes in gene expressions related to incretin synthesis or GH‐IGF‐1 signaling pathways, which were largely unaffected by the postnatal nutrition. Only *IRS1* was consistently upregulated by the HCHF diet in duodenum. This gives rise to the question, whether jejunal growth and digestive function predominantly is regulated by availability of nutrients in the lumen?

In conclusion, we found that late gestation overnutrition in a precocial experimental animal model increased and shifted digestive capacity for carbohydrates toward the jejunum, which can contribute to explain a predisposition in HIGH for development of hyperglycemia upon exposure to the HCHF diet in postnatal life. Late gestation undernutrition predisposed for ileal angiogenesis and carbohydrate and fat hyperabsorptive capacity, but this only became expressed upon subsequent exposure in early postnatal life to a mismatching obesogenic, high‐fat diet. It is intriguing to speculate that these findings can contribute to explain a higher susceptible in LOW individuals toward development of metabolic disorders, such as hypercholesterolemia and the metabolic syndrome. Early postnatal exposure to a high‐fat diet induced a tremendous longitudinal growth and hence increased digestive capacity of particularly the jejunum, although postnatal impacts on gene expression for angiogenic and absorptive markers, were observed almost exclusively in the duodenum.

This study thus demonstrates that the duodenum and ileum overall had similar responses to prenatal malnutrition, whereas postnatal nutrition targeted duodenum and the jejunum. Two questions arise, namely how absorptive capacity in the ileum is determined in fetal life, and which role the duodenum may play in postnatal growth and development of the jejunum?

## CONFLICT OF INTEREST

None.

## Supporting information



Figure S1

Figure S2
