# Supplementary figures and images for: Prenatal over‐ and undernutrition differentially program small intestinal growth, angiogenesis, absorptive capacity, and endocrine function in sheep
Source: Physiol Rep. 2020 Jun 29;8(12):e14498. doi: 10.14814/phy2.14498 (PMC7322502; doi:10.14814/phy2.14498)

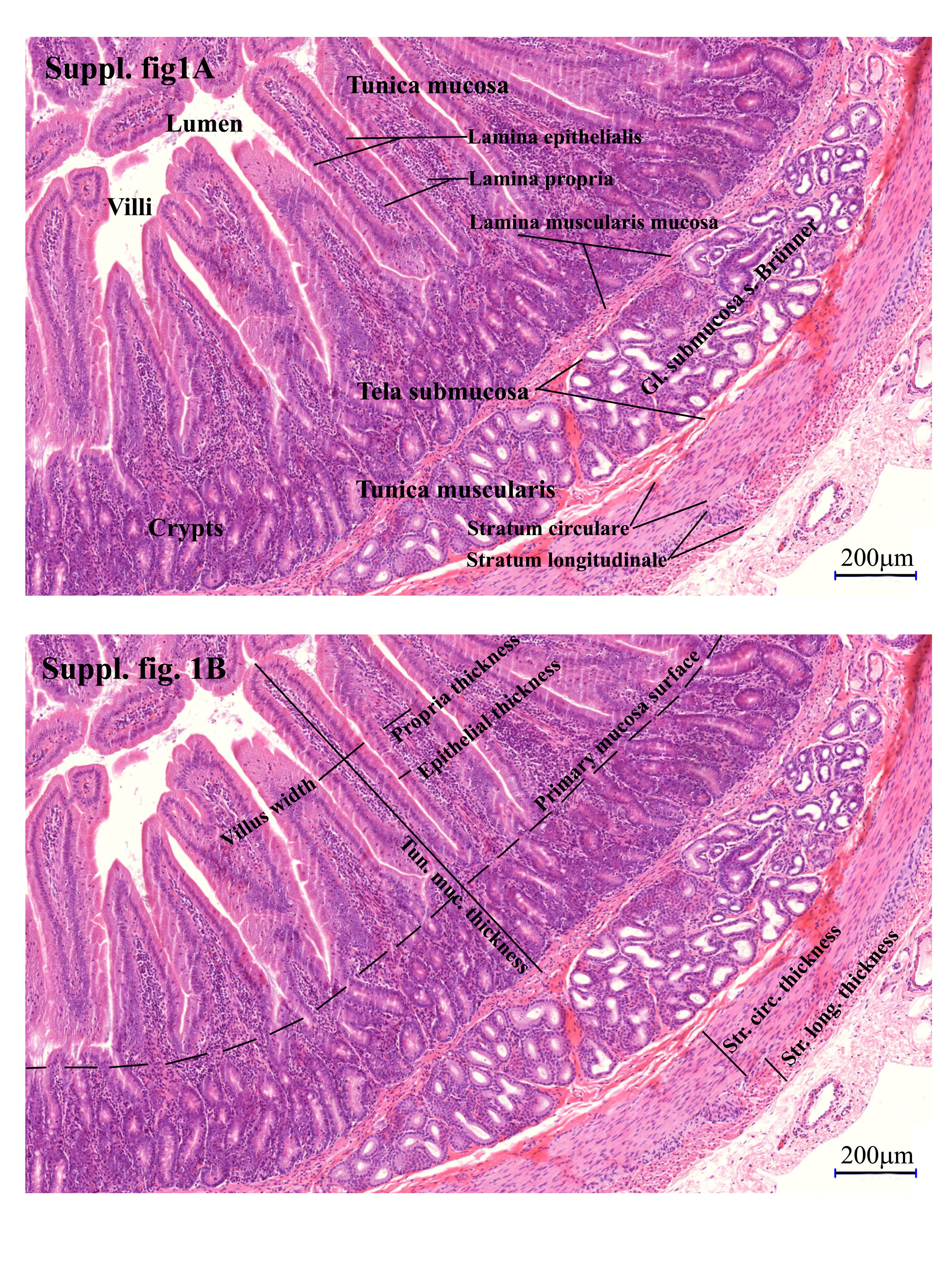

Supplement: Supplementary file 1 — Figure S1 [file PHY2-8-e14498-s001.jpg]

GGT (U/L)

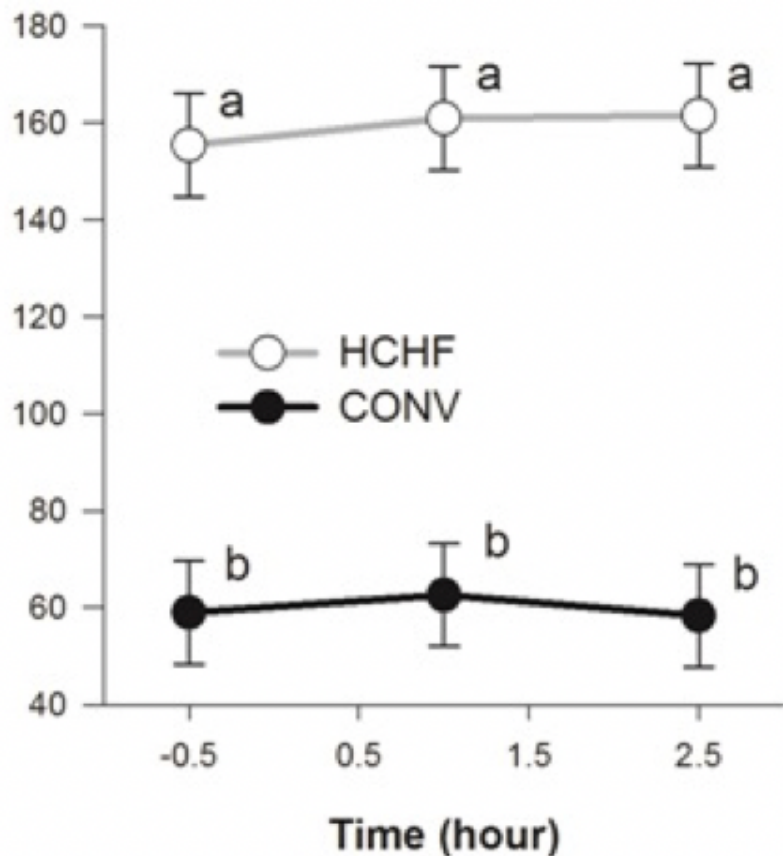

LambDiet:  $p < 0.0001$

LambDiet\*Time:  $p = 0.009$

Supplement: Supplementary file 2 — Figure S2 [file PHY2-8-e14498-s002.pdf]
